# Are highly active and aggressive multiple sclerosis the same entity?

**DOI:** 10.3389/fneur.2023.1132170

**Published:** 2023-03-03

**Authors:** Jorge Correale, Carolina A. Rush, Andrés Barboza

**Affiliations:** ^1^Departamento de Neurología, Fleni, Buenos Aires, Argentina; ^2^Instituto de Química y Fisicoquímica Biológicas (IQUIFIB), Universidad de Buenos Aires-CONICET, Buenos Aires, Argentina; ^3^Department of Medicine-Neurosciences, Ottawa Hospital Research Institute, University of Ottawa, Ottawa, ON, Canada; ^4^Departamento de Neurologia, Hospital Central de Mendoza, Mendoza, Argentina

**Keywords:** multiple sclerosis, aggressive multiple sclerosis, highly active multiple sclerosis, treatment of highly active MS, treatment of aggressive MS

## Introduction

Multiple sclerosis (MS) is a chronic inflammatory demyelinating disease of the central nervous system, leading to neurodegeneration. Advances in imaging and neuropathology have shown that neuroaxonal damage and loss are present from the disease onset. MS is characterized by heterogeneous symptoms, disease course, and outcomes among patients and within the same patient over time. Therefore, different clinical phenotypes have been recently defined based on disease activity—clinical relapse rate and imaging findings—and disease progression (1). In addition to these different phenotypes, the relapsing or progressive disease may, in turn, vary in the severity of signs and symptoms, frequency of relapses, rate of worsening, residual disability, and functional impairment (2). In this context, a subgroup of patients presents with a more aggressive course characterized by the accrual of permanent disability within a short period of time, often with frequent disabling relapses, incomplete recovery, and highly active magnetic resonance imaging (MRI) activity, despite the use of disease-modifying therapies (DMTs). The features observed in this subgroup of patients are referred to as “aggressive MS” or “highly active MS.” However, no consensus has been reached on their definition or therapeutic approach (3). Furthermore, the term “malignant MS” has been occasionally used to describe the most extreme variant of aggressive MS, in which patients exhibit a rapidly progressive course, leading to a significant disability in multiple neurological systems. These patients generally present a monophasic disease with little clinical recovery, resulting in death within a brief period of time (e.g., Marburg's disease and Schilder's disease). Therefore, this label should be used with caution (4).

## Aggressive MS vs. highly active MS

Some studies consider aggressive MS and highly active MS as part of the same spectrum with different degrees of severity, while other authors regard them as different entities (3, 5). However, aggressive and highly active MS share features such as (a) frequent relapses, sometimes with incomplete recovery, and (b) high T2 lesion load on MRI studies and multiple gadolinium (Gd)-enhancing lesions.

While there is no perfect definition of aggressive MS (Table 1), some researchers have described it as an Expanded Disability Status Scale (EDSS) score of 6 reached within 5 years of the MS onset (6, 7), while other studies have defined it as an EDSS of >6 within 10 years of the disease onset (8–10). The group from British Columbia expanded the definition of aggressive MS to include three subgroups based on the EDSS score and progression time to secondary progressive MS (SPMS) as follows: patients with aggressive MS 1 (AMS1) who reached an EDSS of >6 within 5 years of the MS onset, patients with AMS2 who reached an EDSS of >6 by the age of 40, and patients with AMS3 who had SPMS within 3 years of a relapsing onset (5). Of note, many of these definitions are too restrictive and reflect a trade-off between sensitivity and specificity, thus failing to identify high-risk patients who may benefit from higher efficacy treatments, despite their aggressiveness (11).

**Table 1:** Proposed clinical, demographic, and imaging features associated with aggressive and highly active patients with MS.

	**Aggressive MS**	**Highly active MS**
Demographics	Male sex	No specific subset
	Age at onset > 40 years	
	African/American or Hispanic ethnicity	
Relapses	Defined by frequency and severity	>2 relapses in 1 year in naïve patients
	>2 relapses in 1 year in naïve patients	>1 relapse/year in treated patients
	>1 relapse/year in treated patients	Poor recovery from the first 2 relapses
	>1 point EDSS or >2 points in any functional system	>3 relapses within 2 years of disease onset
	Multifocal	
	Incomplete recovery	
	Motor, cerebellar, or sphincter involvement	
MRI findings	High T2 lesion burden	>2 Gd+ lesions at onset or during early follow up
	>20 T2 lesions at disease onset	Ongoing Gd-enhancing lesions despite MS therapy
	>2 Gd+ lesions	Increasing T2 lesion burden despite MS therapy
	>2 Gd+ lesions at disease onset	
	T1 black holes	
	Infratentorial and/or spinal cord lesions	
	Early cortical atrophy	
	Cortical and deep gray matter atrophy	
	Smoldering lesions	

Similarly, highly active MS also lacks a precise definition. The most important features include a high relapse frequency and high radiological burden of Gd-enhancing lesions, which provide evidence of a highly inflammatory form of MS (Table 1). Nevertheless, a consensus is yet to be reached on the cutoff points for the number of relapses or the number of new T2 or Gd-enhancing lesions required, and these thresholds will differ between untreated patients and those receiving DMTs. An ongoing MRI activity, despite a seemingly stable clinical course, is associated with poor prognosis and disability accrual over time. Therefore, patients with a high burden of new T2 lesions or Gd-enhancing lesions or both should be considered highly active MS patients and treated proactively, even if they are clinically silent.

## Prognostic factors

Studies have identified poor clinical and para-clinical prognostic factors in patients with aggressive and highly active MS, which may ultimately render appropriate treatments. Risk factors include demographic and clinical characteristics, MRI activity, and other biomarkers. Natural history cohort studies have demonstrated that men with MS reach disability milestones faster and in larger numbers than women (12, 13). However, other studies have found no association between sex and future disability (14). A late MS onset may represent an additional poor prognostic factor for exhibiting developing aggressive forms. Indeed, patients with the disease onset after the age of 40 years reach disability milestones faster than those with the onset in their twenties and are more likely to have progressive disease features and less likely to have relapses (12). By contrast, pediatric patients with MS have higher annualized relapse rates than adult patients with MS, which suggests a highly inflammatory disease (15). Although the pediatric population has more relapses than adults, children also have a faster recovery (16). Nevertheless, some pediatric patients with MS can become disabled early and experience an aggressive form of disease resulting in early neurologic and cognitive disability (17). Notably, age and sex appear to be independent determinants for the choice of initial DMT in patients with the breakthrough disease (18). In turn, ethnicity has been reported as an additional predictor for patients with aggressive MS, with African American and Hispanic patients showing higher disability rates than Caucasian populations (19, 20).

Clinical characteristics that predict the risk of patients with aggressive MS include the multifocal onset, frequent relapses with incomplete recovery—particularly with motor, cerebellar, or sphincter involvement—early cognitive dysfunction, and poor response to steroids.

In both patients with aggressive and highly active MS, the contribution of relapse activity or disease progression is difficult to establish. However, studies have shown that relapses with partial recovery in the first 2 years of the disease accelerate the accumulation of early disability, while the impact of future relapses in these patients as the disease progresses is minimal (21). Of note, the accumulation of disability can occur as relapse-associated worsening (RAW) and as progression independent of the relapse activity (PIRA) (22–24), which indicates that disability may accumulate even when the relapse activity is seemingly under control. These observations underlie disease progression—which is already relevant in the earliest phases—and suggest that MS is a continuum in which RAW and PIRA occur from the onset (23). PIRA is critically determined by age, being more frequent in older patients (23). Furthermore, the presence of PIRA suggests an unfavorable long-term prognosis, mainly if it occurs early in the course of the disease (25).

Moreover, specific MRI features also associated with poor prognosis—mainly upon early presentation—include T1 black holes at the disease onset, T2 or Gd-enhancing lesion burden, early atrophy, and lesions in the brainstem or spinal cord (7, 22, 26). In line with pathological reports (27), recent studies demonstrated that chronic active lesions identified in 7T (28–30) and 3T (28, 31) susceptibility MRI are associated with more aggressive disease and ongoing tissue damage, which trigger earlier disability. Some of these factors are not necessarily observed with the first clinical episode and must be identified at the onset of the disease or during follow-up in the RRMS phenotype (7).

In addition to MRI, other biomarkers may be necessary to identify patients who are at risk to develop early and rapid disability. The elevated levels of neurofilaments in serum (sNfL) have been associated with the disease activity, including the number of relapses, Gd-enhancing lesions, and paramagnetic rim MRI lesions. Therefore, sNfL levels could represent a potential biomarker in predicting MS disease activity and progression (32). However, sNfL are elevated in neurodegenerative disorders other than MS and are also confounded by age and body mass index. Other approaches have assessed the combination of sNfL and markers of B cell activity. For instance, the combination of cerebrospinal fluid (CSF) CD20^+^/CD14^+^ ratio and NfL levels in CSF and/or serum have been shown to identify patients at risk of rapid disease progression (33).

Aging-associated features vary considerably across individuals. Given the importance of the age of MS onset, the evaluation of biological age can provide crucial information regarding disease prognosis (34). Measuring telomere length represents a good alternative; indeed, short telomeres have been associated with more rapid disability accumulation regardless of age (35). Alternatively, assessing DNA methylation patterns associated with the risk of aging-related diseases may indicate biological age, thus predicting MS outcomes (34).

## Therapeutic implications

Early use of high-efficacy DMTs, rather than the traditional escalation treatment, is more effective at reducing inflammation and provides a window of opportunity as soon as the patient is diagnosed with MS. This strategy may maximize the potential for preventing disability progression (36–38). In addition, patients showing an inadequate subtherapeutic response to DMTs may be offered a second window of opportunity, as a low threshold to switch therapies at the beginning of disease activity may also help prevent future disability (39). Overall, the choice of the most appropriate DMT must be guided by the prognostic factors of each individual, and the final choice should be consensually made with patients after an adequate risk-benefit evaluation (18, 38). Patients with aggressive or highly active MS, however, have narrow windows of opportunity and should therefore be promptly treated with high-efficacy drugs or switch different drugs with a different mechanism of action (7, 38–40). Although the absence of clear definitions for aggressive or highly active MS limits therapeutic decisions, future biomarkers may help better and more quickly identify and stratify this group of patients. Unfortunately, no data exist from prospective clinical trials that specifically address patients with aggressive or highly active MS, while suggestive evidence is available from data derived from *post hoc* analysis or subgroups of patients in MS clinical trials in which different definitions are used (40). However, the possibility of carrying out conventional clinical trials is limited. As a result, pragmatic clinical trials that include subsets of aggressive or highly active patients with MS and assess the efficacy of a specific therapeutic intervention in the real world may prove beneficial (41).

## Discussion

No universally accepted definitions are available for aggressive and highly active MS, and no clear elements are available to allow us to distinguish between the two (10, 40). In other words, patients with rapid disease progression still need to be identified based on prognostic factors in the early stages of the disease (aggressive MS) and the continuous assessment of disease severity (highly active MS) (10). The proposed definitions are hindered by the need for retrospective analysis of the disease course or extended prospective evaluation. Perhaps the main difference between these two entities is a predominantly inflammatory underlying phenomenon in highly active MS, which evokes heightened disease observed in the short term; in turn, both inflammatory and neurodegenerative components, two processes occurring in parallel, account for aggressive MS, which determines more severe permanent disability (42). This difference could have prognostic implications as high-efficacy therapies could modify the long-term prognosis for patients with highly active MS—though not for patients with aggressive MS.

Strikingly, new oligodendrocyte generation has been recently observed in the normal-appearing white matter in a subset of individuals with very aggressive disease, which demonstrates an inherent potential to boost oligodendrogenesis in these patients (43). These findings are in contrast with MRI evidence of chronic active/slowly expanding lesions, which show a demyelination core with axonal transection and an inflammatory demyelinating edge characterized by iron-laden microglia/macrophages (28). It has been hypothesized that patients with chronic active lesions have microglia-specific checkpoint dysregulation (44), which might lead to excessive microglial activation or reduced microglial ability to return to homeostasis (45). These observations, as well as additional damage to uninjured tissue often seen in chronic active lesions, might partly explain remyelination failure, despite the generation of new oligodendrocytes.

We hope this point of view stimulates further discussion on the mechanisms that regulate these MS phenotypes and advances clinical trials incorporating this group of patients to precisely define the best treatment paradigms.

## Author contributions

JC contributed to the conceptualization of the article. All authors drafted and reviewed the manuscript.
